# A huge renal cyst mimicking ascites: a case report

**DOI:** 10.1186/1756-0500-7-39

**Published:** 2014-01-15

**Authors:** Omar Riyach, Mustapha Ahsaini, Karim Tazi, Mohammed Fadl Tazi, Soufiane Mellas, Jalal Eddine El Ammari, Abdelhak Khallouk, Mohammed Jamal El Fassi, Moulay Hassan Farih

**Affiliations:** 1 Department of Urology, University Hospital Center, Hassan II, FES, Morocco; 2 Faculté de médecine et de pharmacie de Fès, Route de Sidi Harazem FES, BP: 1893 –Km 2.200, FES, Morocco

**Keywords:** Renal cyst, Ascites, Ultrasonography, Computed tomography

## Abstract

**Background:**

Renal cysts are common in old patients, and usually remain untreated. Giant renal cyst measuring more than 15 cm in diameter and containing more than 1500 mls of serous fluid are rarely seen. We report a case of a 75-year-old man with a giant right renal cyst.

**Case presentation:**

A 75-year-old man presented with a five years history of suprapubic pain, abdominal distension. He had no urological symptoms. Physical examination revealed a distended abdomen with shifting dullness. Routine hematology, biochemistry, and serum tumor markers were within normal limits. Erroneously diagnosed as ascites on ultrasonographic examination. Abdominal paracentesis of supposed ascites was performed. The diagnosis of giant renal cyst was finally made by Computed tomography (CT) and patient underwent continuous percutaneous catheter drainage with negative pressure, whereby 8 liters of fluid were removed with negative cytology. Subsequent Computed tomography after 6 months revealed disparition of the cysts, and the patient remained asymptomatic.

**Conclusion:**

Giant renal cysts are uncommon; we conclude that the CT remains the best exam in patients evaluated for giant renal cyst. This to the best of our knowledge is the largest renal cyst in the medical literature. Studies are needed with particular attention to the factors associated with renal cyst enlargement.

## Background

Renal cysts are acquired lesions of the kidney [1,2]. They occur commonly in the renal cortex, although the etiology is yet to be fully established, it is believed that they originate from the diverticulum of the distal convoluted tubule [3]. Most benign renal cysts are asymptomatic and require no treatment; they are commonly an incidental ultrasound finding. They can cause a variety of clinical symptoms when they are sufficiently large. It is generally believed to be a harmless anomaly. However, cases of huge renal cysts have been very rarely reported [4]. So we report a unique case of a 75‒ year old male who presented with a massive abdominal distension which was noticed twenty years ago and has increased progressively till presentation to our department. The uniqueness of this case not only lies with the large size but also with the absence of any signs or symptoms apart from the gross abdominal distension.

## Case presentation

**Figure 1:**
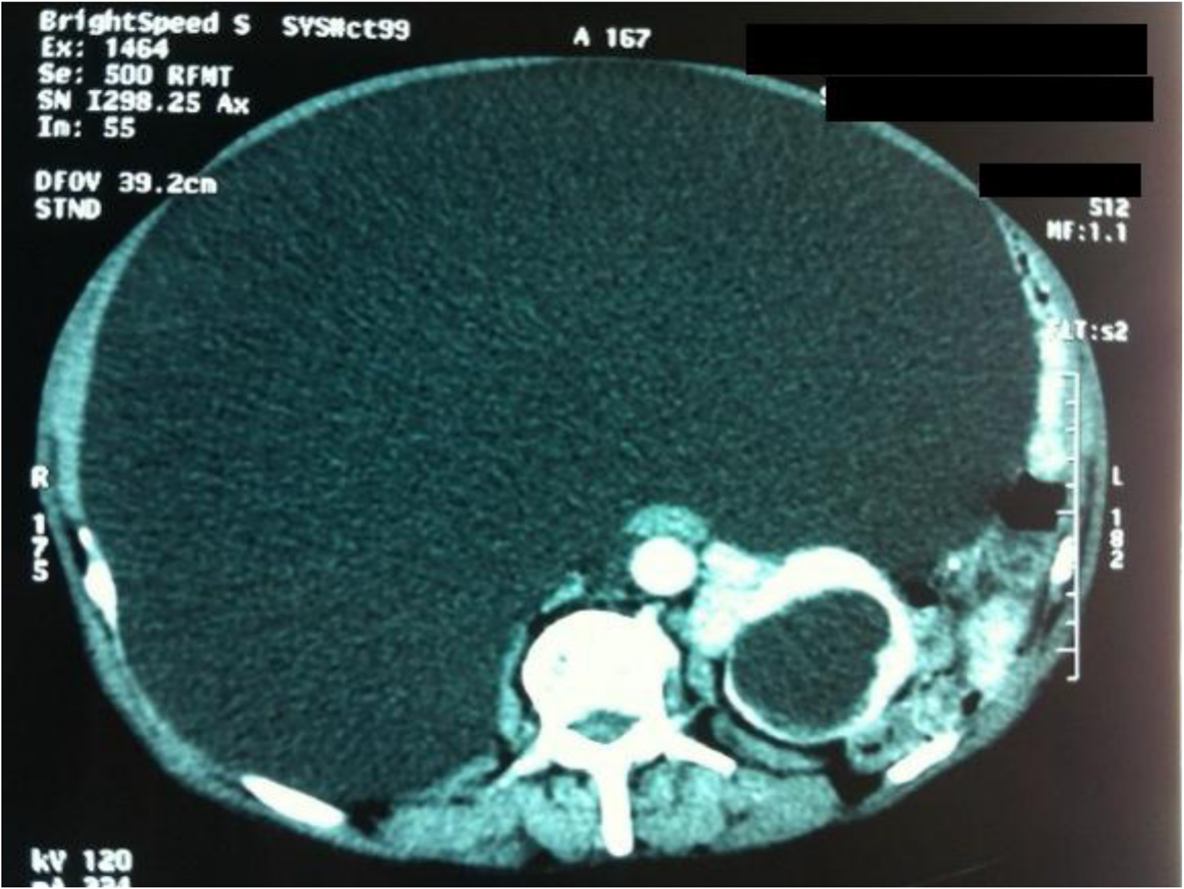
Computed tomography of abdomen revealing bilateral simple renal cysts with an exceptionally giant sized renal cysts measuring 35 × 32 × 22 cm on the right kidney.

**Figure 2:**
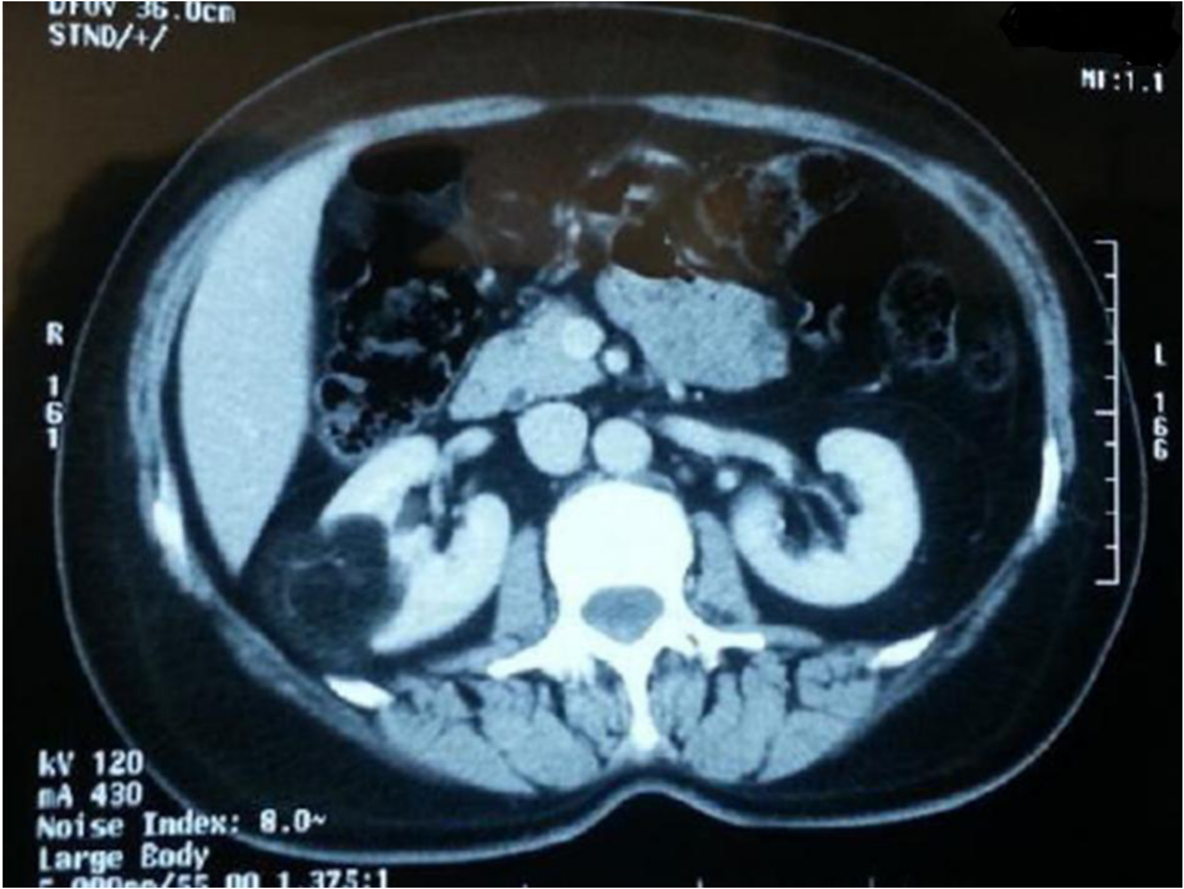
Subsequent computed tomography after six months revealing quasi-normal aspect of kidneys.

A 75-year-old patient was admitted to our department of urology for three years persisting anorexia, progressive weight loss, dehydration and indeterminate significant abdominal distension. Patient was treated for pulmonary tuberculosis, arterial hypertension and hypercholesterolemia. He had no urological symptoms. Physical examination revealed a normal blood pressure, a ballotable mass arising from beneath the costal margin, extending across the midline and into the pelvis. The abdomen was painless on palpation and percussion, the abdominal wall was tough and distended, the prostate was benign. Routine hematology, biochemistry, and serum tumor markers were within normal limits. Voluminous ascites was described on ultrasonographic examination of abdomen. An abdominal paracentesis was performed and gained about 2000 ml of roily brownish fluid without any complications. Fluid’s laboratory examination concluded to transudate with negative microbiological tests and no cytological evidence of malignancy. Computed tomography of his abdomen revealed bilateral simple renal cysts with an exceptionally giant sized renal cysts measuring 35 × 32 × 22 cm on the right kidney (Figure 1). The size of the left renal cysts was 6 cm. The cysts had oval shape, good acoustic enhancement, no internal echoes, and sharply marginated smooth walls. The right giant cyst was shifting the kidney to the midline. The kidney parenchyma was preserved. Treatment consisted of continuous percutaneous catheter drainage with negative pressure on the right kidney, and simple aspiration with sclerotherapy on the left kidney; 8 liters of fluid were removed with negative cytology. The patient reported rapid weight loss of 6 kg and restoration of appetite within days; there were no major post-operative complications. Subsequent Computed tomography after six months revealed quasi-normal aspect of the cysts (Figure 2), and the patient remained asymptomatic.

## Discussion

Renal cyst is one of the most common benign lesions of the kidney and occurring at a frequency greater than 50% over the age of 50 years, and up to 2/3 of population have a cyst detectable on CT scan by the age of 80 years [5]. They are occasionally large enough to be palpable on routine clinical examination or by the patient himself. However, majority of the cysts are < 2 cm in size [6]. They may occur well within a kidney or on its surface, usually oval or round in shape, they have a smooth outline lined by a single layer of flattened epithelium and filled with a transudate‒like clear or straw colored fluid [7]. The etiology of these cysts is unknown. Neonatal renal cysts are described in association with posterior urethral valves [8-10] in contrast valves have not been reported with cysts presenting in older children. It is likely that this obstruction is etiological and that these cysts have developed from calyceal diverticula in utero. The pathogenesis of cysts presenting at a later stage is less clear. If developmental, they may represent abnormalities of earlier generations of uriniferous tubules persisting as cystic collections [11]. If acquired, they may represent renal tubular obstruction secondary to focal ischaemia and inflammation, factors that can produce epithelial cysts experimentally [12]. Giant renal cysts measuring more than 15 cm is an extremely rare occurring pathology [13]. Cases presenting simply with progressive abdominal distension can lead to a misdiagnosis, such as obesity, or ascites as we have reported in our case in which the cyst was enough giant to be visible on inspection and to cause shifting of pelvicalyceal system to other side of midline. Ultrasonography is a helpful examination. It is appreciated for its noninvasiveness, availability and no radiation stress. On the other hand, it is known for its operator-sensitivity, but it still gives misguiding information, as shown in our case report. We focused on erroneously diagnosed ascites and noted a few cases, but none of them described any giant renal cyst imitating ascites. Nonpancreatic pseudocyst [14], giant ovarian cysts [15], cysts of echinococcal [16] and omental [17] origin were misinterpreted as ascites. Giant renal cysts were mistaken for palpable gallbladder [18] and even for obesity [19]. Renal cysts can be treated by percutaneous aspiration with or without injection of sclerosants [20,21], percutaneous marsupialization, and open surgery and, most recently, by laparoscopic surgery with transperitoneal or retroperitoneal access. In our case, we have chosen continuous percutaneous catheter drainage with negative pressure given the simultaneous diagnostic and therapeutic aspects of cyst drainage. This type of a giant cyst has rarely been reported in any medical literature although brown et al., [22,23] reported a giant renal cyst measuring 25 cm in greatest diameter presenting with massive abdominal distension. Our case to the best of our knowledge is the largest renal cyst in the medical literature, and the second case of giant renal cyst imitating ascites. The authors concluded that continuous percutaneous catheter drainage with negative pressure approach shortens the overall operating time and avoids complications and demerits of chirurgical access. In our case laparoscopic approach was not considered feasible.

## Conclusion

Giant renal cysts mimicking ascites are uncommon. Ultrasonography remains the first step exam in patients evaluated for such pathology. However, it is necessary to be aware that in a minority of cases results may be misleading for clinicians as we have reported in our case. It is a reflection of the impact of CT scan that it has increased our awareness, refined our diagnostic process, guided our treatment and may ultimately allows us to study the natural history of these lesions. Further studies need to be carried out with particular attention to results comparison between continuous percutaneous catheter drainage with negative pressure versus aspiration with sclerotherapy for giant renal cyst management.

## Consent

Written informed consent was obtained from the patient for publication of this manuscript and accompanying images. A copy of the written consent is available for review by the Editor-in-Chief of this journal.

## Competing interests

Authors declare that they have no competing interests.

## Authors’ contribution

OR, MA: the principal authors, major contributions in writing the manuscript. KT, MFT, SM, JE, AK, MJE, and MHF: analyzed and interpreted the patient data and the reviews of the literature. All authors read and approved the final manuscript.
